# Patient satisfaction with humanistic nursing in Chinese secondary and tertiary public hospitals: a cross-sectional survey

**DOI:** 10.3389/fpubh.2023.1163351

**Published:** 2023-08-30

**Authors:** Yilan Liu, Fengjian Zhang, Chunyan Guan, Bing Song, Haixin Zhang, Mo Fu, Fang Wang, Chenxi Tang, Huiling Chen, Qingfeng Guo, Ling Fan, Xinfeng Hou, Hongxia Wang, Bing Wu, Geyan Shan, Hongmei Zhang, Feifei Yu, Xiaoping Lou, Hongzhen Xie, Ying Zhou, Gendi Lu, Xia Xin, Shaoshan Pan, Shujie Guo

**Affiliations:** ^1^Department of Nursing, Union Hospital of Tongji Medical College, Huazhong University of Science and Technology, Wuhan, Hubei, China; ^2^Department of Otorhinolaryngology Head and Neck Surgery, Union Hospital, Tongji Medical College, Huazhong University of Science and Technology, Wuhan, Hubei, China; ^3^Department of Orthopedic, Qilu Hospital of Shandong University Dezhou Hospital, Dezhou, Shandong, China; ^4^Department of Nursing, Henan Provincial People's Hospital, Zhengzhou, Henan, China; ^5^Department of Nursing, Jingzhou Central Hospital, Jingzhou, Hubei, China; ^6^Department of Nursing, Laibin People's Hospital, Laibin, China; ^7^Department of Nursing, Nanchong Central Hospital, Nanchong, Sichuan, China; ^8^Heart Center of Henan Provincial People's Hospital, Central China Fuwai Hospital, Central China Fuwai Hospital of Zhengzhou University, Zhengzhou, Henan, China; ^9^Department of Nursing, The Fourth Affiliated Hospital of Harbin Medical University, Harbin, Heilongjiang, China; ^10^Department of Nursing, Shengjing Hospital of China Medical University, Shenyang, Liaoning, China; ^11^Department of Nursing, Luohe Central Hospital, Luohe, Henan, China; ^12^Assisted Reproductive Centre, Henan Provincial People's Hospital, Zhengzhou, Henan, China; ^13^Institute of Psychology and Behaviour, Henan University, Kaifeng, Henan, China; ^14^Outpatient of International Medical Center, Henan Provincial People's Hospital, Zhengzhou, Henan, China; ^15^Department of Nursing, The First Affiliated Hospital of Zhengzhou University, Zhengzhou, Henan, China; ^16^Department of Health Medicine, People's Liberation Army General Hospital of Southern Theatre Command, Guangzhou, Guangdong, China; ^17^School of Nursing, Guangzhou Medical University, Guangzhou, Guangdong, China; ^18^Department of Shuguang Hospital Affiliated to Shanghai University of Traditional Chinese Medicine, Shanghai, China; ^19^Department of Nursing, First Affiliated Hospital of Xi'an Jiaotong University, Xi'An, Shanxi, China; ^20^Department of Nursing, General Hospital of Southern Theatre Command, Guangzhou, Guangdong, China; ^21^Department of Outpatient, Henan Provincial People's Hospital, Zhengzhou, Henan, China

**Keywords:** Chinese, hospital patients, patient satisfaction, humanistic nursing, national survey, influencing factors

## Abstract

**Background:**

Humanistic care pertains to the abilities, attitudes, and behaviors central to patient-centered care, contributing to patients' sense of safety and wellbeing. This study aimed to assess the satisfaction of patients with humanistic nursing care in Chinese secondary and tertiary public hospitals.

**Methods:**

A national cross-sectional survey was conducted across 30 provinces and 83 hospitals in China. Patient satisfaction with humanistic care was assessed using the Methodist Health Care System Nurse Caring Instrument (NCI), which encompasses 20 items across 12 dimensions. Each item was rated on a 7-point Likert scale, yielding a total score of 140. Multiple linear regression analysis was employed to identify factors associated with patients' satisfaction.

**Results:**

Moderate satisfaction (mean score 91.26 ± 13.14) with humanistic nursing care was observed among the 17,593 participants. Factors significantly associated with patient satisfaction included age, hospital type, presence of children, educational attainment, place of residence, family monthly income, and medical insurance type.

**Conclusion:**

The study findings highlight the importance of tailored interventions, evidence-based practice guidelines, and patient-centered care in improving patients' satisfaction with humanistic nursing care. Continuous emphasis on nursing education and professional development is crucial for enhancing humanistic care and patient satisfaction.

## 1. Introduction

Humanistic care refers to the abilities, attitudes, and behaviors of patient-centered care. It enables patients to feel cared for and respected during the caring process, which can inculcate a sense of safety and security and help patients achieve physical, spiritual, and sociocultural wellbeing (1–4). The concept of humanistic care was initially proposed in the 1970s by Madeleine Leininger, an American nursing scholar, and was subsequently developed and expanded upon by Jean Watson, who established the theoretical underpinnings of nursing humanism (5). Humanistic nursing is vital for patients, is an important aspect of patient satisfaction, and is an essential component of quality nursing care (6). The lack of humanistic nursing directly affects patient recovery, reduces the quality of medical services and patient satisfaction, and can lead to friction between nurses and patients (7–12). Compared to Europe and American countries, China's humanistic nursing started later but has developed rapidly. In the 1990s, some Chinese universities began to offer courses on humanistic care in nursing at different times, but they have not yet been fully integrated into the national curriculum. The “Development Plan for Nursing in China (2011–2015)” released in 2011 proposed to “highlight the characteristics of the nursing profession, increase the proportion of psychology, humanities, and social sciences in the curriculum, and enhance awareness of humanistic care”. Since then, humanistic care in nursing has been promoted nationwide in China. In recent years, nursing schools and medical institutions across all levels in China have introduced training programs in humanistic nursing care. However, despite these efforts, the overall awareness and development of humanistic care among Chinese nurses remain low (13–15). A cross-sectional study conducted on the humanistic care competencies of clinical nursing staff in Central China showed that the overall care competencies of Chinese nursing staff were poor and fell significantly below those of nursing staff in Europe and the United States (16). Patient satisfaction with humanistic nursing is not only a measure of nurses' humanistic care practice but also a basis for nursing managers to test the effectiveness of humanistic care and an important reference for the development of humanistic care practice standards (17, 18). Nationwide cross-sectional survey data on inpatient satisfaction with humanistic care in China is lacking at present. An extensive literature search identified only two studies on the above topics, and both were single-center studies with small sample sizes, which did not adequately reflect the overall situation of humanistic care practices in China (19, 20). The Humanistic Care Committee of the China Life Care Association conducted the first national multi-center survey on patient satisfaction with humanistic nursing care in China. The objective of this study was to provide a reference for the nationwide evaluation of humanistic nursing care practices and support nursing managers in formulating intervention measures.

## 2. Methods

### 2.1. Study design

**Figure 1:**
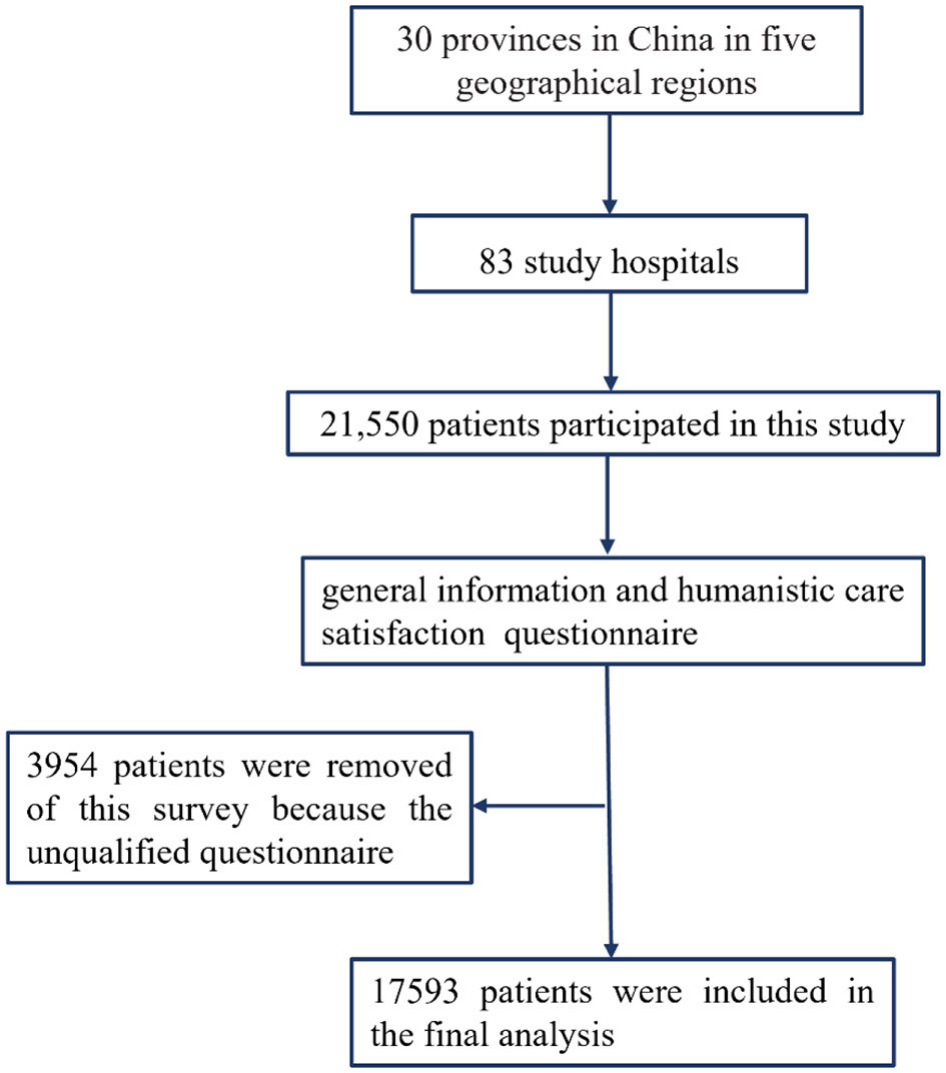
Flowchart of participants throughout the study.

The medical institutions surveyed in this study were all members of the Humanistic Care Professional Committee of the Chinese Association for Life Care. The study population was determined using a multi-stage stratified sampling method. The data collection lasted 45 days (1 July 2022–15 August 2022). During the first stage of the study, we selected 22 provinces, four autonomous regions, and four municipalities based on the regional distribution of China Life Care Association's hospital members. The sample size from each provincial unit was determined according to its population proportion in relation to China's total population. The sampling frame excluded Hong Kong, Macao, Taiwan, and the Xizang autonomous regions. In the second stage, the number of secondary and tertiary hospitals to be included in the study was determined for each province by considering the population distribution of each city within their jurisdiction. The surveyed hospital managers were members of the National Humanistic Nursing Special Committee. In the third stage, the questionnaires were administered to a random sample of patients at the locations of discharge checkout in the selected hospitals until the desired survey sample size was reached. To ensure a representative sample, the number of participants per hospital was determined based on the hospital's size, while demographic factors such as age, gender, and diagnostic indicators were considered to minimize confounding biases. Figure 1 presents a schematic diagram illustrating the process employed in this study.

### 2.2. Survey participants

The primary formula used for determining the sample size in this study was as follows: n = u${\;}_{\text{α}/2}^{2}\pi \left(1-\pi \right)/\delta^{2}$, where u_α/2_ = 1.96 for α = 0.05, π represents the anticipated patient satisfaction with humanistic nursing (which was 70% in this study), and δ denotes the admissible error (which was set at 10% for this study). Using this formula, the theoretical sample size was calculated as 90, with an additional 10% added to account for any potential loss of participants during the study. Taking into account the variation in the number of hospital inpatients and to make the data more representative, we decided to enroll a minimum of 350 patients from each tertiary hospital and 100 patients from each secondary hospital to ensure adequate representation. The inclusion criteria were as follows: (1) all participants were stable inpatients; (2) all participants or their legal guardians were older than 18 years; and (3) study participants or their legal guardians provided written informed consent. The exclusion criteria were as follows: (1) patients unable to complete the cognitive assessments required for the trial and (2) patients without smartphones or who were unable to answer the questionnaire using a smartphone. The total number of patients interviewed for this study was 21,550. However, to control for selection bias, a final sample of 17,593 participants was included in the analysis after excluding incomplete or invalid questionnaires.

### 2.3. Survey instruments

The survey comprised two sub-questionnaires developed and used after approval by the research team based on a comprehensive literature review (21, 22). The general information questionnaire included the patient's hospital, sex, age, marital status, number of children, education level, place of residence, family monthly income, whether it was the first visit, time of visit, type of medical insurance, department visited, area of visit, and whether surgery was performed. The Humanistic Care Satisfaction Scale was assessed using the Methodist Health Care System Nurse Caring Instrument (NCI), developed by the Nursing Care Quality Control Council of the Houston Health Care System in 2000 as part of the Humanistic Care Satisfaction questionnaire (21). This questionnaire is the most commonly used instrument for assessing the quality of care and consists of 20 items covering 12 dimensions: care coordination, competence, teaching/learning, emotional support, respect for individuality, physical comfort, availability, helping/trusting relationships, patient/family involvement, physical environment, spiritual environment, and outcomes. The items are reflective of concepts from the caring literature and caring theory of *Watson* and others, as well as items that are familiar areas of assessment on other instruments. Each item includes seven answers corresponding to a 7-point Likert scale. For each item, “*seldom or rarely*”, “*often or frequent*”, “*always or almost always*”, and “*does not apply*” were scored from 1 to 7, resulting in a total score of 140. A higher score indicates greater satisfaction with nurses' care.

After obtaining authorization from the original authors, we embarked on a comprehensive adaptation and reliability testing process for this study. Our team meticulously employed a multi-faceted scientific approach to develop, localize, and validate the questionnaire's reliability, feasibility, and acceptability. This robust process encompassed an extensive literature review, patient cognitive interviews, input from a diverse range of stakeholders (including healthcare regulators, hospital managers, doctors, nurses, and patients), psychometric analyses, pilot tests conducted across three provinces, small-scale multi-disciplinary expert consultations, and field tests. To ensure the feasibility and acceptability of the tool, we analyzed missing item response percentages, reviewed interviewer-reported acceptability, and assessed the time and ease of administration. The internal consistency and reliability of each dimension were evaluated using Cronbach's α coefficients and inter-subscale correlations. The Chinese version of the NCI questionnaire demonstrated excellent reliability in this study, with an overall Cronbach's α of 0.982. Moreover, the helping/trusting dimension obtained a Cronbach's α of 0.943, the respect for individuality dimension had a Cronbach's α of 0.907, the patient/family involvement dimension reached a Cronbach's α of 0.937, the emotional support dimension achieved a Cronbach's α of 0.895, and the care coordination dimension garnered a Cronbach's α of 0.908. For the remaining dimensions, which consisted of single items, Cronbach's α coefficients could not be calculated. Overall, the questionnaire's strong reliability is well-supported within the context of our study, underscoring a coherent, logical, and methodically sound research design.

### 2.4. Data collection

To ensure high-quality data collection and analysis, the Chinese Association for Life Care's Humanistic Care Professional Committee initiated and coordinated this study, which was conducted using the Questionnaire Star Platform. After obtaining hospitals' consent to participate, the head nurse of the investigation department in different hospitals and departments across the country received the questionnaire, and its purpose was explained. The head nurses were trained to ensure standardized administration procedures, covering the purpose of the study, its significance, the target population, and the method of completing the questionnaire. All questionnaire items were submitted after completion, and the questionnaire was completed anonymously and independently. To ensure the validity of the questionnaire completion, each IP address could only be submitted once. Data entry was performed by two independent researchers to ensure data accuracy, and missing data and poor-quality questionnaires were excluded.

Rigorous quality control measures were implemented to ensure the high quality of data collection and analysis. First, the head nurses provided standardized training to all nurses who administered the questionnaires, ensuring consistent procedures in participant selection and response recording. Second, data cleaning and verification were conducted to remove invalid or incomplete responses, and discrepancies were resolved through consultation with relevant nurses. A random sample of questionnaires was selected for re-interviews to verify data accuracy and consistency.

### 2.5. Ethical consideration

The protocol of this study was reviewed and approved by the Ethics Committee of Tongji Medical College, Huazhong University of Science and Technology, with an ethics approval number of 2022S161. The developers permitted the use of NCI questionnaires in this study. Approval for data collection was obtained from the directors of the institutions that collected the data. Informed consent was obtained from all participants before they completed the questionnaire.

### 2.6. Statistical methods

An Excel spreadsheet was exported from the Questionnaire Star platform to create the original database. After excluding invalid questionnaires, the data were imported into SPSS version 25.0. The two-person cross-check method was used to reduce errors and ensure the accuracy of the data. Continuous variable distributions were characterized by means and standard deviations, while categorical variable distributions were summarized using frequency counts. Relevant indicators were initially analyzed using univariate methods, and those exhibiting differences were incorporated into a multivariate logistic regression analysis to identify risk factors. Differences were deemed statistically significant at a *P* < 0.05.

## 3. Results

### 3.1. Comparison between included and excluded patients

The total number of patients interviewed for this study was 21,550. Of these, 17,593 patients were included with valid questionnaires, and 3,957 patients were excluded with invalid questionnaires. The primary reasons for their exclusion were the presence of full scores, zero scores, and incomplete responses, as encountered during our survey process. There were no statistically significant differences between the general information of the included and excluded patients, as shown in Table 1.

**Table 1:** Comparison of demographic characteristics between included and excluded patients.

**Project**		**Included patients**	**Excluded patients**	**χ^2^-value**	***P*-value**
Gender	Male	9,313	2,019	0.623	0.625
	Female	8,280	1,938		
Age	18–34	5,639	1,420	0.856	0.421
	35–59	7,728	1,658		
	≥60	4,226	879		
Hospital	Secondary hospital	3,196	1,031	1.368	0.281
	Tertiary hospital	14,397	2,926		
Marital status	Married	14,215	2,778	0.924	0.322
	Single	2,755	812		
	Divorced or separated	160	129		
	Widowed	463	238		
Children	No child	5,160	432	0.349	0.727
	1 child	8,532	1,575		
	≥2 children	9,061	1,950		
Education attainment	Primary school the following	3,321	671	0.637	0.225
	Junior high school	3,278	834		
	High school/technical secondary school	3,661	876		
	College	3,268	783		
	Bachelor degree or above	4,068	793		
Occupation	Farmer	4,063	962	0.768	0.216
	Worker	1,629	403		
	Military person	172	20		
	Leader	1,103	233		
	Employed	3,526	878		
	Self-employed	1,307	300		
	Freelance	2,038	327		
	Retired	2,078	365		
	Student	969	238		
	Other	708	231		
	Place of residence	City	6,632			1,325	0.581	0.337
		Towns	5,915			1,168		
		Rural	5,046			1,464		
	Family monthly income (Yuan)	< 3,000	4,918			1,230	0.968	0.252
		3,000– < 5,000	6,053			1,259		
5,000– < 8,000		3,528	782					
>8,000		1,583	362					
>10,000		1,511	324					
Department visited	Internal medicine	8,523	1,753	0.383	0.713
	Surgical	5,986	1,343		
	Obstetrics and gynecology	1,816	630		
	Pediatric	628	114		
	Intensive care medicine	473	82		
	Other	167	35		
Medical insurance type	Own expense	1,269	310	0.472	0.582
	Town healthcare	9,826	1,865		
	City healthcare	4,583	1,186		
	Provincial healthcare	1,085	365		
	Commercial insurance	163	43		
	Public expense	263	62		
	Other	404	126		
Region	Central	4,337	959	0.347	0.621
	East	5,361	968		
	Northeast	1,910	435		
	West	3,858	821		
	Northwest	2,127	774		
First time visited	No	7,855	1,758	0.645	0.519
	Yes	9,738	2,199		
Surgical patient	No	10,524	2,128	1.304	0.121
	Yes	7,069	1,829		

### 3.2. Humanistic care satisfaction scores of patients with diverse characteristics

**Figure 2:**
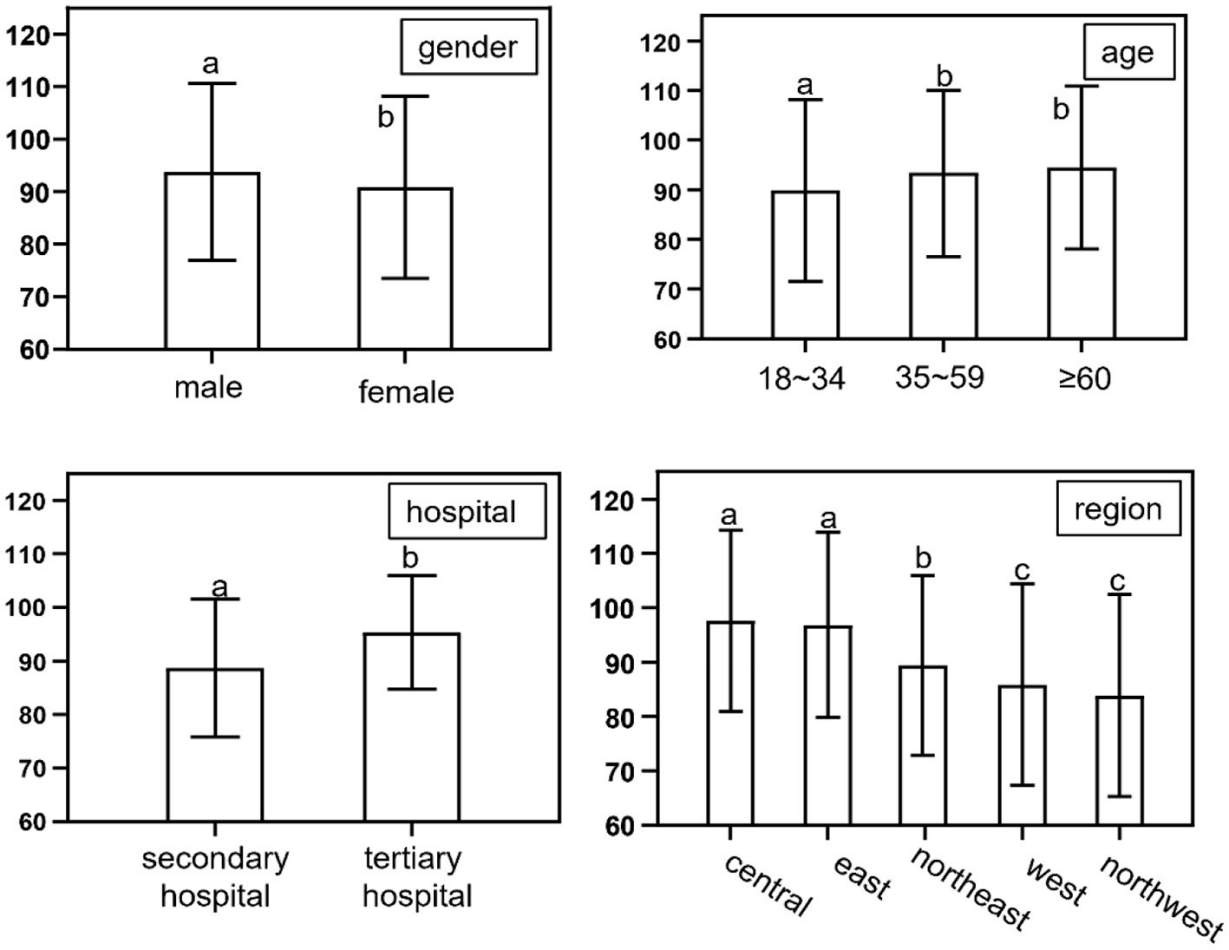
Comparison of humanistic caring satisfaction scores with general demographic features. PS. Differences were considered not significant where there was a single identical letter mark, and significant where there were different letter marks.

The final analysis included participants from 30 provinces: 1,910 (10.9%) from Northeast China, 5,361 (30.5%) from Eastern China, 4,337 (24.7%) from Central China, 2,127 (12.1%) from Northwest China, and 3,858 (22.0%) from Western China. Participants' ages ranged from 18 to 90 years, with a mean age of 46.27 ± 17.09 years. The sample consisted of 9,313 (52.94%) male participants and 8,280 (47.06%) female participants. The Humanistic Care Satisfaction Scale had a full score of 140, and the overall mean satisfaction score of patients' humanistic care in this study was 91.26 ± 13.14. Table 2 shows the results of the comparative analysis of humanistic care satisfaction scores among patients with various characteristics. Statistically significant differences were observed in humanistic care satisfaction scores based on sex, age, marital status, presence of children, educational attainment, occupation, place of residence, family monthly income, department visited, medical insurance type, region, and whether patients were surgical (*P* < 0.05). No significant differences in nursing care satisfaction scores were found among first-time visiting patients (*P* > 0.05). Figure 2 depicts the subgroup analysis according to the general demographic features, emphasizing a considerable increase in satisfaction scores for humanistic nursing care among male patients compared to female patients. Additionally, the satisfaction scores of nursing care showed a gradual increase with age and a significant improvement in tertiary hospitals relative to secondary hospitals. The highest levels of nursing satisfaction were observed in Central China, followed by Eastern China, with scores steadily decreasing from the eastern to the northwestern regions. Notably, the lowest nursing satisfaction scores were identified in Northwest China.

**Table 2:** Comparison of humanistic caring satisfaction scores with different demographic characteristics.

**Project**		**Numbers**	**Score (*x* ±*s*)**	**Statistics**	***P-*value**
Average score		17,593	91.26 ± 13.14	-	-
Gender	Male	9,313	93.79 ± 16.86	4.623^*a*^	0.035
	Female	8,280	90.85 ± 17.36		
Age	18–34	5,639	89.91 ± 18.30	44.250^*b*^	0.021
	35–59	7,728	93.38 ± 16.76		
	≥60	4,226	94.45 ± 16.36		
Hospital	Secondary hospital	3,196	88.68 ± 12.82	10.339^*a*^	0.021
	Tertiary hospital	14,397	95.38 ± 10.56		
Marital status	Married	14,215	93.43 ± 16.93	7.225^*b*^	0.022
	Single	2,755	92.43 ± 17.96		
	Divorced or separated	160	90.62 ± 19.80		
	Widowed	463	88.77 ± 17.76		
Children	No child	5,160	87.47 ± 17.83	12.349^*b*^	0.027
	1 child	8,532	93.81 ± 16.82		
	≥2 children	9,061	94.03 ± 17.12		
Education attainment	Primary school the following	3,321	83.58 ± 16.89	3.137^*b*^	0.025
	Junior high school	3,278	85.54 ± 16.84		
	High school/technical secondary school	3,661	87.18 ± 17.27		
	College	3,268	92.51 ± 17.55		
	Bachelor degree or above	4,068	93.25 ± 17.20		
Occupation	Farmer	4,063	94.25 ± 16.35	17.768^*b*^	0.016
	Worker	1,629	94.95 ± 16.01		
	Military person	172	93.24 ± 20.49		
	Leader	1,103	90.16 ± 16.78		
	Employed	3,526	93.07 ± 17.38		
	Self-employed	1,307	91.73 ± 18.08		
	Freelance	2,038	91.37 ± 17.99		
	Retired	2,078	94.38 ± 16.35		
	Student	969	92.21 ± 17.97		
	Other	708	86.48 ± 18.86		
Place of residence	City	6,632	89.74 ± 16.92	17.581^*b*^	0.037
	Towns	5,915	92.99 ± 17.40		
	Rural	5,046	92.34 ± 17.37		
Family monthly income (Yuan)	< 3,000	4,918	92.43 ± 17.53	8.968^*b*^	0.020
	3,000– < 5,000	6,053	88.24 ± 17.14		
	5,000– < 8,000	3,528	89.98 ± 16.61		
	>8,000	1,583	94.07 ± 16.69		
	>10,000	1,511	95.06 ± 17.54		
Department visited	Internal medicine	8,523	93.32 ± 17.00	17.383^*b*^	0.013
	Surgical	5,986	94.12 ± 16.60		
	Obstetrics and gynecology	1,816	90.58 ± 18.28		
	Pediatric	628	90.23 ± 18.53		
	Intensive care medicine	473	88.41 ± 17.87		
	Other	167	84.79 ± 16.80		
Medical insurance type	Own expense	1,269	86.55 ± 18.93	15.472^*b*^	0.012
	Town healthcare	9,826	93.55 ± 16.99		
	City healthcare	4,583	93.49 ± 16.78		
	Provincial healthcare	1,085	94.29 ± 15.99		
	Commercial insurance	163	89.39 ± 17.33		
	Public expense	263	95.80 ± 17.28		
	Other	404	86.06 ± 18.03		
Region	Central	4,337	97.60 ± 16.65	36.147^*b*^	0.021
	East	5,361	96.83 ± 17.04		
	Northeast	1,910	89.21 ± 16.49		
	West	3,858	85.90 ± 18.57		
	Northwest	2,127	83.90 ± 18.57		
First time visited	No	7,855	93.16 ± 17.04	0.645	0.519
	Yes	9,738	93.29 ± 17.22		
Surgical patient	No	10,524	93.05 ± 17.25	2.304^*a*^	0.021
	Yes	7,069	93.52 ± 16.97		

a is t-value;

b is F-value.

### 3.3. Factors associated with humanistic care satisfaction scores

The multiple linear regression analysis (Table 3) revealed significant associations between various factors and humanistic care satisfaction scores. Age (β = 0.605, SE = 0.026), hospital (β = 0.663, SE = 0.239), presence of children (β = 0.265, SE = 0.032), education attainment (β = 0.591, SE = 0.171), place of residence (β = 0.274, SE = 0.027), family monthly income (β = 0.338, SE = 0.238), and medical insurance type (β = 0.353, SE = 0.321) were found to have significant positive correlations with humanistic care satisfaction scores. In contrast, gender (β = −0.862, SE = 0.051), marital status (β = −0.231, SE = 0.131), department visited (β = −0.274, SE = 0.241), region (β = −0.393, SE = 0.061), and surgical patient status (β = −0.266, SE = 0.083) displayed significant negative correlations with humanistic care satisfaction scores. The R^2^-value for this analysis was 0.662, and the adjusted R^2^-value was 0.495.

**Table 3:** Multiple linear regression analysis of the factors associated with humanistic care satisfaction scores.

**Variable**	**β**	**SE**	***P***	**R^2^**	**Adjusted R^2^**
				0.662	0.495
Gender	−0.862	0.051	0.021		
Age	0.605	0.026	0.011		
Hospital	0.663	0.239	0.009		
Marital status	−0.231	0.131	0.025		
Children	0.265	0.032	0.018		
Education attainment	0.591	0.171	0.021		
Occupation	0.383	0.124	0.136		
Place of residence	0.274	0.027	0.023		
Family monthly income	0.338	0.238	0.036		
Department visited	−0.274	0.241	0.022		
Medical insurance type	0.353	0.321	0.016		
Region	−0.393	0.061	0.043		
Surgical patient	−0.266	0.083	0.032		

## 4. Discussion

This study surveyed 83 hospitals across 30 provinces in China, and while the sample may not be entirely representative of all public hospitals in the country, the findings did provide some insight into the average level of humanistic care in Chinese secondary and tertiary institutions. The full score of the Humanistic Care Satisfaction Scale was 140, and the mean score for patient satisfaction with humanistic care was calculated as 91.26 ± 13.14, indicating a moderate level of humanistic care in China. To the best of our knowledge, this was the first national cross-sectional survey concerning humanistic care using the Methodist Health Care System Nurse Caring Instrument (NCI). A cross-sectional survey of 66,348 hospital patients in England using the Hospital Consumer Assessment of Healthcare Providers and Systems (HCAHPS) scale showed that approximately 77% of patients were satisfied with the care services they received. The reasons for this are poor professional nurse staffing and poor hospital work environments (23). In Nigeria, a similar survey of 185 patients showed that approximately 60.4% of patients were satisfied with the care they received (24). In the United States, a cross-sectional study of 409 hospitals using the HCAHPS scale in four states showed that 60% of patients were satisfied with their primary nurses (25). In contrast to other countries' patient satisfaction scores, the average patient satisfaction scores in China, using the NCI scale, remained at the same level. These results have been boosted considerably by the Chinese government's emphasis on patient satisfaction. Governments at all levels in China have developed several regulations and rules to support patient-centered care and provide humanistic care to patients. Nursing schools at all levels in China have launched humanistic care courses to cultivate and enhance nursing students' awareness and abilities in providing humanistic care. Medical institutions at all levels have successively developed humanistic care wards, training, and scientific research. The Humanistic Nursing Professional Committee of China developed the “Expert Consensus on the Practice Specification for Humanistic Nursing Care in Hospitals” and the “Management Specification for Humanistic Nursing Care in Hospital Wards” in 2021 and filled the gap of the lacking consensus and standards for humanistic nursing care in China (26, 27). Since then, expert consensus and standards on humanistic care in sub-specialties have been consecutively developed.

The study results revealed significant differences in nursing care satisfaction scores among patients with different sex, age, marital status, presence of children, educational attainment, occupation, place of residence, family monthly income, department visited, medical insurance type, regions, and whether they underwent surgery. Female patients exhibited lower satisfaction scores for humanistic care compared to male patients, which might be due to female patients' sensitivity to the spiritual, cultural, and emotional aspects of humanistic care (28). Patients aged 60 or older reported the highest satisfaction with nursing care, potentially related to their life experience, which enables them to understand healthcare professionals' psychological thoughts and empathize with their support (29). Higher satisfaction with humanistic care was observed in patients with higher family incomes, living in urban areas, and who had provincial health insurance or public expenses, consistent with findings from studies on inpatient satisfaction in public hospitals (30). Higher educational attainment led patients to expect increased demands for medical service quality and humanistic care (31). For low-income patients, medical expenses represented a substantial burden, resulting in lower humanistic care satisfaction (31). Patients from different provinces also displayed variability in satisfaction, with patients in Western provinces showing lower patient care satisfaction. A survey on the humanistic care abilities of medical professionals in Western China indicated a low capacity for nursing workers in the region (32). Economic development forms the basis for a robust medical system and resource allocation, necessitating increased support for economically underdeveloped regions (33). Simultaneously, medical quality acts as a protective factor for patient satisfaction, making improving medical quality crucial for enhancing patient satisfaction. Various consultation departments and surgical patient status can also impact nursing care satisfaction. Departments such as intensive care units and outpatient clinics, which maintain high-intensity work environments, may experience challenges in implementing caring behaviors, thereby affecting patients' perceptions of nursing care (34, 35).

The multiple linear regression analysis identified several risk factors significantly associated with patient satisfaction with humanistic care. These factors included age, hospital type (secondary or tertiary), presence of children, educational attainment, place of residence, family monthly income, and medical insurance type. Older patients demonstrated higher satisfaction with humanistic nursing due to their accumulated life experiences and deeper understanding of healthcare professionals' psychological thoughts, fostering empathy toward them. In contrast, younger patients might have higher expectations for nursing care, leading to dissatisfaction if expectations are not met. Consequently, addressing different age groups' specific needs and expectations is crucial when tailoring humanistic nursing approaches (36, 37). Patients from tertiary hospitals exhibited higher satisfaction with humanistic care than those from secondary hospitals, possibly due to tertiary hospitals' access to advanced medical resources and expertise, which contribute to higher-quality nursing services and improved patient-centered care. Therefore, investing in humanistic nursing service development and enhancing staff training in secondary hospitals are imperative to address this gap (38, 39). Patients with children, higher education levels, residing in urban areas, and higher family monthly incomes reported greater satisfaction with humanistic care. These factors suggest that patients with better social support and resource access likely have higher expectations for humanistic nursing services. Thus, implementing targeted interventions to address these patient populations' unique needs and concerns is essential. Medical insurance type also emerged as a significant factor affecting patient satisfaction with humanistic care. Patients with provincial health insurance and public expenses displayed higher satisfaction, indicating that medical insurance policies should consider incorporating humanistic care aspects to improve overall patient satisfaction.

The study findings hold several significant implications for nursing practice and management in China. First, nursing managers should recognize humanistic care's importance in improving patient satisfaction and identify and address factors negatively affecting satisfaction scores. Interventions should target specific risk factors such as gender, age, education, occupation, place of residence, and healthcare settings to cater to individual patients' distinct needs, preferences, and expectations. Second, nursing managers should ensure continuous professional development training and education for nurses in humanistic care. This training should focus on developing cultural competence, building rapport, effective communication, empathy, and active listening skills. Third, hospitals and nursing managers should invest in developing evidence-based practice guidelines and strategies for implementing patient-centered care. Organizational policies and practices should promote a culture valuing humanistic care and facilitate its implementation within a supportive environment. Finally, future research should evaluate targeted interventions designed to improve humanistic care provision among different patient populations and identify innovative nursing practice strategies and approaches that can enhance patient satisfaction with humanistic care services.

In conclusion, this study contributes to the existing literature on patient satisfaction with humanistic nursing care in China, particularly among secondary and tertiary public hospitals. The findings highlight the significance of humanistic care in patient satisfaction and provide valuable insights into the associated risk factors. Through the identification and careful management of these risk factors, nursing managers can promote better humanistic care practices and ultimately improve patient outcomes and experiences.

### 4.1. Limitations

This survey has several limitations that should be noted. First, due to constraints in human resources, the survey was unable to cover several remote provinces in China, which may have resulted in biased findings. Additionally, the exclusion of community hospitals may limit the generalizability of the results to the entire Chinese population. To address these limitations, we plan to expand the survey to provide a more comprehensive understanding of healthcare satisfaction in China. Specifically, we will seek additional resources to conduct surveys in the provinces not covered in this study and include more community hospitals to ensure adequate representation across different regions and healthcare institutions. Furthermore, we will refine our survey methodology and process to enhance the accuracy and credibility of the data, with the ultimate goal of generating more objective and comprehensive research findings.

## 5. Conclusion

This national cross-sectional survey discovered moderate satisfaction with humanistic nursing care in Chinese secondary and tertiary public hospitals. Factors significantly associated with patient satisfaction include age, hospital type, presence of children, educational attainment, place of residence, family monthly income, and medical insurance type. These results emphasize the importance of tailored interventions, evidence-based practice guidelines, and patient-centered care. Considering the study's limitations, future research should expand the survey coverage, refine the methodology, and evaluate targeted interventions to improve humanistic care provision among diverse patient populations. Furthermore, continuous emphasis on nursing education, professional development, and innovative nursing practices is vital for enhancing humanistic care and patient satisfaction.

## Data availability statement

The raw data supporting the conclusions of this article will be made available by the authors, without undue reservation.

## Ethics statement

The Ethics Committee of the Henan Province People's Hospital approved the study protocol. The developers permitted the usage of NCI questionnaires in this study. Approval for data collection was obtained from the directors of the institutions that collected the data. Informed consent was obtained from all participants before they completed the questionnaire.

## Author contributions

SG, SP, YL, GL, YZ, HX, and GS: study conception and design. CG, BW, BS, HaZ, MF, FW, CT, HC, QG, LF, XH, HW, GS, HoZ, FY, XL, and XX: acquisition of data. YL, FZ, SP, SG, BW, and HC: analysis and interpretation of data. YL, FZ, SG, SP, and BS: write up the manuscript and critical revision. All authors contributed to the article and approved the submitted version.
